# Cysteinyl-tRNA Deacylation Can Be Uncoupled from Protein Synthesis

**DOI:** 10.1371/journal.pone.0033072

**Published:** 2012-03-09

**Authors:** Alexandre David, Suman R. Das, James S. Gibbs, Jack R. Bennink, Jonathan W. Yewdell

**Affiliations:** Laboratory of Viral Diseases, National Institute of Allergy and Infectious Diseases, Bethesda, Maryland, United States of America; National Institutes of Health, United States of America

## Abstract

Aminoacyl-tRNA synthetases (ARSs) are critical components of protein translation, providing ribosomes with aminoacyl-tRNAs. In return, ribosomes release uncharged tRNAs as ARS substrates. Here, we show that tRNA deacylation can be uncoupled from protein synthesis in an amino acid specific manner. While tRNAs coupled to radiolabeled Met, Leu Lys, or Ser are stable in cells following translation inhibition with arsenite, radiolabeled Cys is released from tRNA at a high rate. We discuss possible translation independent functions for tRNA^Cys^.

## Introduction

Aminoacyl-tRNA synthetases (ARSs) catalyze the attachment of the 20 canonical amino acids to their cognate tRNAs. Since ribosomes poorly discriminate between amino acid homologs, ARSs play an essential role in minimizing errors in amino acid incorporation into proteins. Recent findings suggest that modifications in ARS specificity can alter decryption of the genetic code, adding an unexpected twist to the function of these ancient enzymes at the very center of cellular function [1], [2]

ARSs can be classified by different means. Based on the architecture of their catalytic site -thought to reflect the primordial enzymes - they can be divided into two classes of 10 ARSs each [3] including 6 subclasses total (grouping enzymes having the closest relationship). Recently, we classified ARSs into 3 distinct groups, based on their association with ribosomes [4]: “free” ARSs (designated by their single letter amino acid code, ARS, CRS, GRS, HRS, NRS, SRS, TRS, VRS, WRS, YRS), ARSs forming the Multi-aminoacyl-tRNA Synthetase Complex (MSC: EPRS (ERS and PRS are present in a single protein), DRS, IRS, KRS, LRS, MRS, QRS, and RRS), and FRS. FRS tightly associates with ribosomes regardless of translation activity, while the MSC associates with polysomes, and the free ARSs loosely associate with polysomes.

The translation-dependent association of ARSs with polysomes builds on the work of Deutscher and colleagues indicating that translation is a channeled process likely to be highly compartmentalized to enable mRNAs to be translated under optimal conditions [5]. It is now clear that polysomes partition into multiple intracellular locales. It is likely that the functions of ARSs and tRNA species utilized are intricately regulated to increase the efficiency of protein biogenesis.

Adding to the complexity, ARSs have a number of defined alternative functions, hardly surprising for such ancient and abundant (∼10^7^ copies/cell) proteins. Here we characterize the functions of representative ARSs and their cognate tRNAs in cells under normal and stressed conditions.

## Results and Discussion

**Figure 1:**
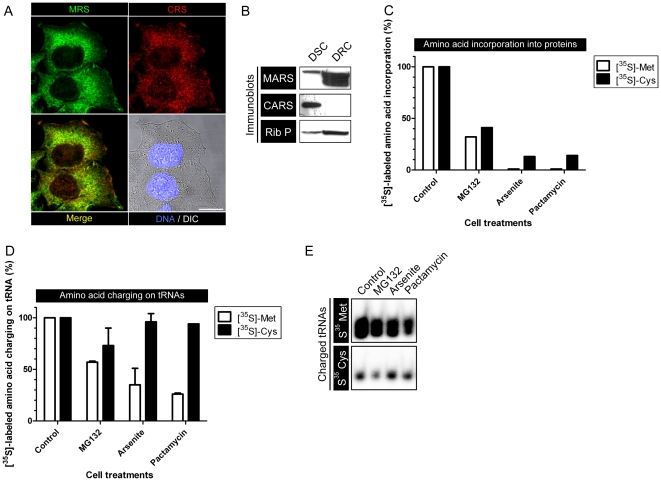
MRS and CRS define two distinct cytosolic compartments. A. Immunofluorescence staining of HeLa cells. MRS (green) and CRS (red) only partially co-localize. Bar scale: 10 µm. B. Immunoblot of sequential HeLa cells extractions. Cells were first extracted with digitonin (DSC: digitonin soluble compartment), then washed and extracted with NP-40 (DRC: digitonin resistant compartment). Membranes were blotted for MRS, CRS and ribosomal P proteins. C. HeLa cells were pre-treated for 2 h with MG132 or for 30 min with either arsenite or pactamycin. Then cells were labeled with [^35^S]-Cys or [^35^S]-Met. Half of each sample was used to extract total proteins and cpm were counted. 100% cpm was determined using untreated HeLa cells. For [^35^S]-Met: 100% = 982785 cpm. For [^35^S]-Cys: 100% = 371828 cpm. D. The other half of each sample from “C” was used. Total RNAs were extracted and cpm counted. 100% cpm was determined using untreated HeLa cells. For [^35^S]-Met: 100% = 288248 cpm. For [^35^S]-Cys: 100% = 69183 cpm. E. The same samples used in “D” were loaded on acid gels to visualize either [^35^S]-Met-tRNAs or [^35^S]-Cys-tRNAs. The two bands for [^35^S]-Met-tRNAs correspond to the mitochondrial tRNA^Met^ and the nuclear encoded tRNA^Met^.

In the course of studying the phenomenon of MSC-mediated Met-misacylation, we compared the properties of the two sulfur amino acid ARSs, MRS and CRS, both members of the Ia ARS subclass. While MRS and CRS exhibit a similar distribution pattern by immunofluorescence, they demonstrate markedly different detergent solubilities. CRS is readily released from cells by mild detergent (digitonin), while ∼90% of MRS is retained by cells, possibly due to MSC association with ER-complexed polysomes (Fig. 1A and 1B) [4], [6].

In exploring the charging status of tRNA by pulse labeling cells with [^35^S]-Met or [^35^S]-Cys following various perturbations, we found a number of surprising differences in their behavior. First, although the specific activities of the amino acids are similar according to the supplier, [^35^S]-Met is incorporated at ∼10-fold higher levels in tRNA after pulse labeling (Fig. 1D and 1E). Since Met and Cys are similarly represented in proteins, this suggests a fundamental difference in the contribution of extracellular vs. intracellular pools for Met vs. Cys tRNA acylation.

Second, we treated cells with inhibitors that block protein synthesis directly (pactamycin, which inhibits translation initiation [7]), or indirectly by inducing stress with MG132 (which inhibits proteasomes), or the oxidizing agent sodium arsenite [8]. These treatments inhibited incorporation of Met and Cys into proteins in parallel (Fig. 1C) (the small additional amount of Cys incorporation into proteins following translation inhibition is not due to translation, but to post-translational association [9]). As expected from the inhibition of protein synthesis, Met tRNA acylation was greatly inhibited, which we attribute to the limited amount of substrate (unconjugated Met-tRNA). By contrast, Cys acylation continues unabated in cells with essentially no translation activity. These findings were made by measuring radioactivity in tRNA fractions (Fig. 1D). These data were correlated with tRNA isolated in agarose gels, which provides somewhat less precision and reproducibility than the direct counting method due to additional processing steps (Fig. 1E).

**Figure 2:**
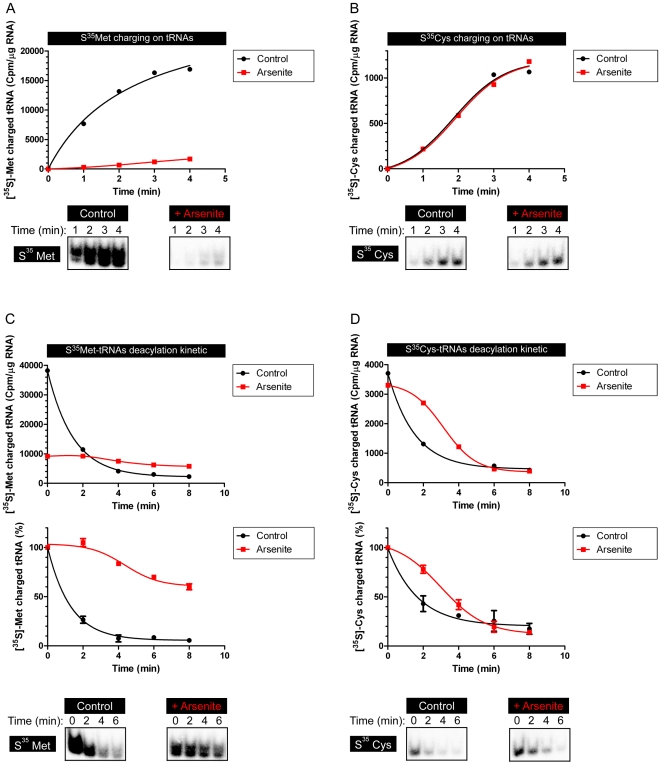
Cys-tRNA deacylation is uncoupled from translation. A. Kinetics of [^35^S]-Met charging on tRNAs with or without pre-treating HeLa cells with sodium arsenite. The same samples were both analyzed by beta counter and loaded on a native acid gel. B. Same as “A” with [^35^S]-Cys charging. C. Kinetics of [^35^S]-Met deacylation on tRNAs extracted from HeLa cells pretreated or not with sodium arsenite. This panel shows two different graph representations: “raw” data using Cpm/µg of total RNA and representing one experiment or calibrated data defining time 0 as 100% of [^35^S]-Met charged tRNAs and averaging 2 distinct experiments. The same samples were both analyzed by beta counter and loaded on native acid gels. D. Same as “A” with [^35^S]-Cys deacylation.

We confirmed disparities that can occur in Met *vs.* Cys tRNA acylation by measuring their on- and off-kinetics in control cells or cells incubated with arsenite to inhibit translation. Met charging was severely depressed, while Cys charging was remarkably unaffected by translational status (Fig. 2A and 2B). Similarly, Met discharging was minimal in arsenite treated cells while Cys discharging was only slightly impeded by arsenite (Fig. 2C and 2D).

**Figure 3:**
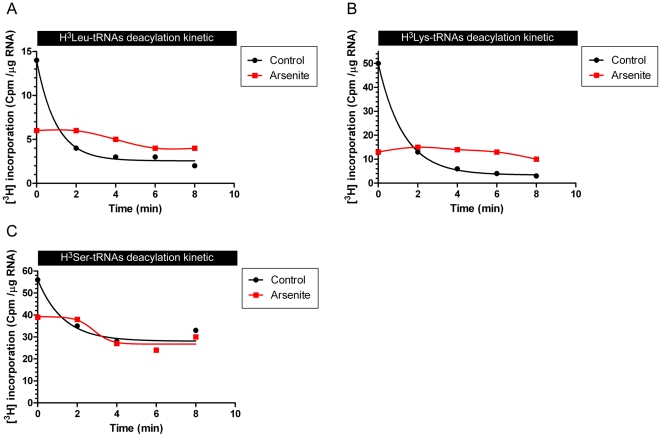
Leu-tRNA, Lys-tRNA and Ser-tRNA deacylation is coupled to translation. A. Kinetic of [^3^H]-Leu deacylation on tRNAs extracted from HeLa cells treated or not with sodium arsenite. B. Same as “A” with [^3^H]-Lys deacylation. C. Same as “A” with [^3^H]-Ser deacylation.

We extended these findings to 3 other radiolabeled amino acids: [^3^H]-Leu, [^3^H]-Lys and [^3^H]-Ser. LRS and KRS are MSC components [10]. SRS, like CRS, is “free” in the cytosol [4]. The deacylation of these 3 amino acids was inhibited by protein synthesis inhibitors (Fig. 3A, 3B and 3C), underlining the specificity of the deacylation mechanism observed for Cys-tRNA^Cys^.

Our findings differ somewhat from Aspen and Hoagland's [11], who also studied the kinetics of tRNA acylation/deacylation in HeLa cells. Leu, Arg, and Ser (as well as Phe, Val, and Lys) were all rapidly released from tRNA when protein synthesis was inhibited by incubation at 15°C or at 30°C in the presence of the chain elongation inhibitor cycloheximide (CHX). The extent to which this is due to differences in temperatures used in the two studies (all of our experiments were performed at 37°C) or inhibitors used (cycloheximide *vs.* aresenite) remains to be determined.

The differential behavior of extracellular [^35^S]-labeled Met and Cys tRNA acylation and incorporation into protein has an immediate practical implication. When using Tran^35^S-label mixtures of Met and Cys to label cells, more than 95% of translationally incorporated radiolabel should represent Met, since Met is present at 5∶1 in the mixture (according to the supplier), and is incorporated ∼10-fold more efficiently into tRNA. Further, the differential behavior of Met- *vs.* Cys-tRNA in the absence of protein synthesis will confound analysis of off-kinetics, particularly if one of the tRNAs is enriched following fractionation. This may contribute to the differential behavior of ER *vs.* cytosol Tran^35^S-labelled- tRNA reported by Stephens et al. [12].

The mechanism underlying Cys-tRNA^Cys^ deacylation remains to be established. One suspect is CRS, since ARSs are generally capable of catalyzing deacylation [13], [14]. Another candidate is PDRX, the human homolog of the bacterial Ybak Cys-tRNA^Cys^ deacylase, hypothesized to be involved in cell growth control [15]. We have been unable to amplify its mRNA in HeLa cells by PCR, suggesting its absence in this cell line. Given the energy dependence of tRNA acylation (one ATP-AMP conversion per cycle), and the expense of synthesizing more CRS than absolutely necessary for protein synthesis, it is likely that Cys deacylation serves a function. Since a significant fraction of cellular Cys is present in glutathione, it is possible that Cys deacylation is linked in some manner to glutathione regulation. Acylated tRNAs are known to participate in post-translational addition of amino acids to proteins. Cys is not documented to modify proteins in this manner, but a number of MHC bound peptides are cysteinylated, consistent with this possibility [16], [17]. On the other hand, cysteinylation should be relatively infrequent, and not account for the substantial off rate of Cys in the absence of translation, fully half the delivery rate for protein synthesis. Staschke et al. [18] reported that tRNA^Cys^, along with several other tRNAs, are deacylated in yeast in response to amino acid starvation, and suggested a possible role for deacylated tRNA^Cys^ in regulating cellular responses to nutritional status.

Research into protein translation has returned to the main stage of cell biology, with progress driven by application of advances in bioinformatics, microscopy, and mass spectrometry. This is timely, as so many basic aspects of translation are poorly understood. It is clear that protein synthesis is more complex than simple decoding. Rather, translation is subtly regulated by mRNA structure, codon usage, modulation of ARS specificity, and is organized spatially to optimize interactions of nascent chains with chaperones and other factors to facilitate protein folding and assembly. Our findings underscore the many unknowns regarding the trafficking of tRNAs in maximizing their efficiency in mediating translation and other competing processes.

## Materials and Methods

### Cells and treaments

HeLa cells (ATCC) were cultured in DMEM (Invitrogen, Carlsbad, CA) supplemented with 7.5% FBS (HyClone Laboratories, Logan, UT), at 37°C, 9% CO_2_. Cells were plated overnight in T75 flasks to yield ∼80% confluence at the start of the experiment.

Drugs were used at the indicated concentrations and times: MG132 (5 µM, 2 h, Sigma), pactamycin (0.5 µM, 30 min), sodium arsenite (500 µM, 30 min, Sigma).

### Cell extractions

HeLa cells were extracted by a sequential detergent extraction protocol based on previous studies [4], [19]. Briefly, cell monolayers were washed with PBS, and incubated with permeabilization buffer (50 mM Tris-HCl pH 7.5, 5 mM MgCl_2_, 25 mM KCl, 100 µg/mL CHX, EDTA-free protease inhibitors (Roche), 10 U/mL RNAse Out (Invitrogen)) containing 0.015% digitonin for 5 min on ice. The supernatant was recovered and cells were washed once with permeabilization buffer. Permeabilized cell monolayers were then solubilized with an equal volume of permeabilization buffer containing 1% NP40 for 5 min on ice. The supernatant (membrane-bound fraction) was recovered, and both DSC and DRC were loaded on a SDS-PAGE gel.

### Immunofluorescence and Microscopy

Immunofluorescence was performed using staining buffer (0.05% saponin, 10 mM glycine, 5% FBS, PBS) as previously described [4]. Following immunostaining procedure, cells were labeled with Hoechst (Molecular Probes). Then, coverslips were mounted with Fluoromount-G (SouthernBiotech). Images were acquired with a Leica TCS SP5 confocal microscope (LAS AF software) and HCX PLAPO 63× objective lenses (Numerical aperture: 1.4). Images were processed with Adobe Photoshop using only the levels and contrast adjustments.

### Antibodies

Mouse anti-SRS and anti-MRS were purchased from Abnova. Human anti-ribosomal P antiserum was from Immunovision.

### Acylated tRNA extraction

∼1×10^7^ HeLa cells per treatment were pulse-labeled with 1 mCi of [^35^S]-Met or [^35^S]-Cys or [^3^H]-Leu or [^3^H]-Lys or [^3^H]-Ser (Amersham or Perkin-Elmer) for 8 min (in a custom DMEM lacking the corresponding amino-acid), washed and resuspended in 500 µl of 300 mM sodium acetate pH4.8 (NaOAc/HOAc)/10 mM EDTA. Acidic pH was chosen to stabilize the aminoacyl linkage. Cells were transferred to tubes containing 1.4 mm ceramic spheres (MP, Solon, OH) and 500 µl of NaOAc/HOAc saturated phenol-chloroform (Perkin-Elmer) and 1% SDS, incubated at 4°C for 30 min with vigorous shaking, and centrifuged at 13,000 rpm for 15 min at 4°C. RNA was precipitated from the aqueous phase using an equal volume of isopropanol, washed with 70% ethanol/30% 50 mM NaOAc/HOAc pH4.8, dried and resuspended in 10 mM NaOAc/HOAc pH4.8/1 mM EDTA.

For deacylation experiments, cells were pulse-labeled for 5 min, washed twice with cold DMEM containing 7.5% FBS and incubated for 2, 4, 6 and 8 minutes with warm DMEM containing 7.5% FBS.

Each sample was diluted in “Optiphase Supermix scintillation liquid” (PerkinElmer) and cpm were counted using a β-counter MicroBeta Trilux (PerkinElmer).

Prism software (GraphPad) was used for analysis and curve fitting.

### Radiolabeled protein extraction and cpm counting

∼1×10^7^ HeLa cells per treatment were pulse-labeled the same way as for tRNA extraction (they were done simultaneously with HeLa cells used for total RNA extraction). Cells were washed twice with cold PBS before being resuspended with 500 µL Lysis Buffer (1% SDS, 150 mM NaCl, 50 mM Tris-HCl pH7.5) supplemented with proteasome inhibitor tablet (Roche) and MG132 (5 µM). Cell lysates were boiled for 20 min at 95°C before being diluted in 1 mL of Neutralizing Buffer (1.5% NP-40, 150 mM NaCl, 50 mM Tris-HCl pH7.5). 2 µL of each sample were applied per spot of 96 filter mat (averaging 6 replicates per condition). After drying at 60°C, the mat was incubated in 10% TCA for 30 min at RT, washed twice in 70% ethanol solution (10 min/wash), dried at 60°C and placed in a scintillation bag with 5 mL of scintillation liquid (PerkinElmer). Radioactivity was quantitated using a β-counter (1450 LSC Trilux, PerkinElmer).

### Acid gel

Native acid gels were made based on a previously published protocol [20]. Briefly, each gel was constituted by 10% acrylamide (National Diagnostic), 7 M urea (Invitrogen) gel, 25 mM acetate buffer, pH 4.8. Each gel was first pre-run in 100 mM tris-acetate pH 4.8 for 1 h. For each condition, 2 µg of total RNA was loaded and run at 4°C for ∼5 h, Then, each gel was vacuum dried and exposed to phosphorimaging plates (Fuji Medicals) for 16–24 h.
